# Screening of Potential HIV-1 Inhibitors/Replication Blockers Using Secure Lentiviral in Vitro System

**Published:** 2011

**Authors:** M.M. Prokofjeva, P.V. Spirin, D.V. Yanvarev, A.V. Ivanov, M.S. Novikov, O.A. Stepanov, M.B. Gottikh, S.N. Kochetkov, B. Fehse, C. Stocking, V.S. Prassolov

**Affiliations:** Engelhardt Institute of Molecular Biology, Russian Academy of Sciences; Volgograd State Medical University; Belozersky Institute of Physico-Chemical Biology, Lomonosov Moscow State University; Research Department Cell and Gene Therapy, Department for Stem Cell Transplantation University Medical Center Hamburg-Eppendorf; Heinrich-Pette-Institute for Experimental Virology and Immunology

**Keywords:** HIV, lentiviral vectors, pseudo-HIV-1 particles, nucleoside reverse transcriptase inhibitors, non-nucleoside reverse transcriptase inhibitors; integrase inhibitors

## Abstract

The development and usage of safe cell systems for testing agents which possess anti-HIV activity is a very important factor in the design of new drugs. We have described in detail a system we designed that is based on lentiviral vectors (Prokofjeva et. al.,*Antiviral Therapy,*in print) for swift and completely safe screening of potential HIV-1 replication inhibitors. The system enables one to test the efficiency of the inhibitory activity of compounds whose action is directed towards either wild-type HIV-1 reverse transcriptase or integrase, or mutant enzymes corresponding to the drug-resistant virus form. Testing results of a number of already known drugs, which correlate well with published data as well as data on newly synthesized compounds, were obtained. Application of this system substantially broadens the possibilities of preclinical anti-HIV drugs testing.

## INTRODUCTION

**Fig. 1:**
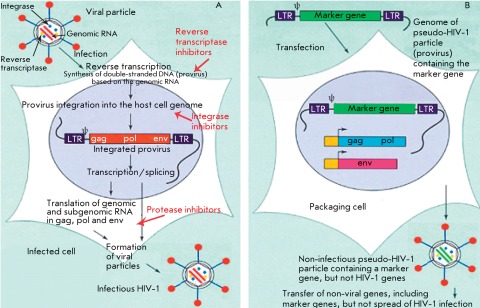
The life cycle of infectious HIV-1 (A) and production of recombinant pseudo-HIV-1 particles in packaging cells (B).

The human immunodeficiency virus type 1 (HIV-1), which belongs to the lentivirus genus of the retrovirus family, is responsible for one of the most common and life-threatening diseases known as the acquired immunodeficiency syndrome (AIDS). According to the World Health Organization (WHO), by the end of 2008, the number of HIV-1-infected people topped 33 million [1]. In 2010, official records put the number of HIV-1-infected people in Russia at 520,000  [2]. It should be noted that in reality the actual number of infected people can be two or even three times higher. It follows from the prognoses of the WHO and non-governmental organizations that even if all the initiatives to control AIDS propagation were implemented and anti-HIV therapy was used, the number of HIV-infected people may still exceed 48 million in the next several years.

Despite great efforts, no efficacious preventive or therapeutic vaccine has as yet been designed. The use of low-molecular inhibitors of different stages of the replicative cycle of the virus remains the only therapeutic approach upon HIV infection. Thus far, approximately 30 substances of varying structures have been designed and certified as anti-HIV drugs. The majority of these substances inhibit three HIV-1 enzymes: reverse transcriptase (RT), integrase, and protease; the so-called fusion blockers (blocker of viral penetration into the cell) were recently added to this list [3]. The simultaneous use of several substances of different types in cases of highly active antiretroviral therapy (HAART) enables to achieve a relatively long-term and noticeable decrease in virus titer in the blood; hence, a patient’s life is prolonged considerably [4, 5]. Nevertheless, all the aforementioned substances have several limitations. Firstly, long-term administration of drugs is required because of the lifetime HIV infection, resulting in the emergence of new mutant forms of the virus, which are resistant to the drugs used and can further spread in the virus population. As a consequence, viral forms that are insusceptible to one or even all classes of the above-listed anti-HIV-1 drugs have been detected in approximately 10% of the U.S. and European patients who had never been exposed to antiretroviral therapy [6]. Secondly, the need for long-term therapy often increases the possibility of adverse effects from antiretroviral agents [7, 8]. Thus, the search for new compounds with anti-HIV-1 activity is an extremely important issue in modern virology and medicinal chemistry. Moreover, it appears necessary to develop new agents that can be both relatively safe for patients and at the same time active towards both the wild-type virus and its drug-resistant forms.

An important stage in the development of new antiretroviral agents is testing their efficacy. Most laboratories involved in the search for new anti-HIV agents do not have the opportunity to work directly with the infectious replication-competent virus. This kind of research, which involves personnel coming into direct contact with the natural virus, can be performed only in certified laboratories that provide conditions that ensure operational safety and have permission to deal with class III hazard infectious substances. In this regard, the development and use of safe cell systems to test antiviral activity is of quite high significance in the design process of new therapeutic agents. Lentiviral vectors, whose functional activity manifests itself as a result of the activity of all HIV-1 enzymes, are of particular interest for expeditious and safe screening of potential inhibitors of HIV-1 replication.

Since the early 1980s, vectors based on simple and complex retroviruses have been intensively used as powerful universal tools, including those for designing efficient transfer systems and for the expression of different genes and interfering RNAs in human and animal cells both *in vitro* and *in vivo* [9–13]. 

Lentiviral vectors have been used in our laboratory, as well as in other laboratories, in order to design safe systems for the screening of inhibitors of wild-type HIV-1 replication [14–18]. These systems are represented by a recombinant lentivirus carrying a fragment of the HIV-1 genome, without the regions that encode virus peptides and contain the gene of a reporter (marker) protein (e.g., green fluorescent protein). Moreover, pseudoviral particles are composed of the enzymes that are required for HIV-1 replication (reverse transcriptase, integrase, and protease), which provides the potential to synthesize a DNA copy of this genome, as well as the possibility to integrate it into the host cell genome via the same mechanism as the one at play in the infectious HIV-1. It is essential that these pseudo-HIV-1 particles can carry coat proteins of HIV-1 or other enveloped viruses (e.g., G-protein from vesicular stomatitis virus) on their surface, depending on researchers’ choice. This provides the possibility of using certain lines of eukaryotic cells (target cells) and sufficiently high infection efficiency. The assembly of HIV-1-like particles occurs in this system according to the modified procedure that was developed for constructing virus-like particles on the basis of the murine leukemia virus that is related to HIV-1 [19] ( *Fig. 1* ). This procedure consists in individual introduction of plasmids containing a) the *gag-pol* gene of HIV-1 that encodes the structural proteins for the formation of the capsid of a viral particle and HIV-1 enzymes, b) the *env* gene that encodes glycoproteins of the HIV-1 envelope or the gene of the envelope protein of another virus, and c) antiviral DNA that encodes the recombinant RNA genome containing the marker gene of the fluorescent protein to the cultivated human embryonic kidney cells (the so-called packaging cells). After all the components listed are introduced into the packaging cells, viral proteins and recombinant RNA ensuring the formation of the HIV-1-like particles that are released into the environment are synthesized in the aforementioned cells. The addition of these particles to the target cells induces the synthesis of the DNA of a provirus that contains a marker gene, whose integration into the target cell genome renders it capable of fluorescing on the recombinant RNA genome in target cells. It should be stressed that the use of plasmid DNAs expressing individual virus-specific proteins enables to construct any variants of pseudo-HIV-1 particles with one or several mutations in any enzyme of viral replication which correspond to the drug-resistant HIV-1 strains. 

Thus far, published investigations still contain an insufficient number of examples of successful use of these systems to study the antiretroviral activity of substances that differ in their nature; this makes it unclear just how universal the described systems are . In this regard, our study mainly endeavoured to verify the adequacy of the cell system proposed for screening potential anti-HIV-1 agents. The activity of a number of inhibitors of HIV-1 reverse transcriptase and integrase were tested, both of which have found application in medical practice and have undergone various stages of laboratory research.

## EXPERIMENTAL

**Cell cultivation**

**Fig. 2:**
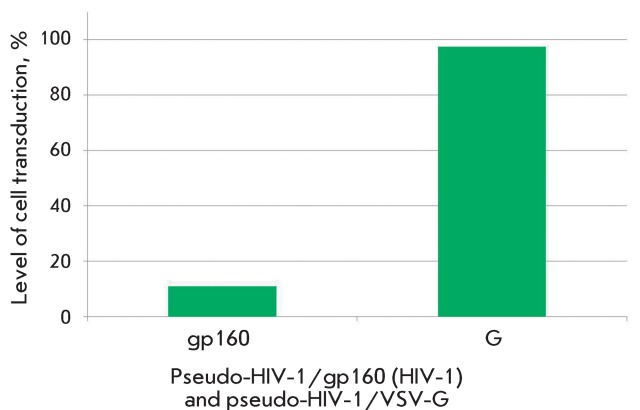
The level of transduction of the Jurkat cell line with pseudo-HIV-1 particles containing glycoprotein gp160  of HIV-1 or glycoprotein G of the vesicular stomatitis virus (VSV-G) as an envelope protein.

**Fig. 3:**
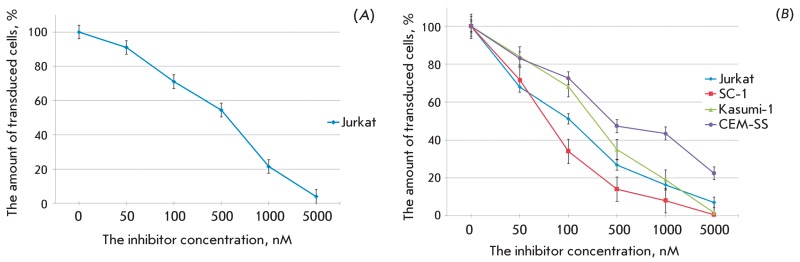
The action of AZT on the transduction efficiency of different cell lines with pseudo-HIV-1 particles containing the envelope protein gp160 (A), or the envelope protein VSV-G (B). The level of transduction is shown with respect to the positive control.

 The following cell lines were used in this study: HEK293 (human embryonic kidney cells), SC-1 (mouse embryonic fibroblasts), Jurkat (human T-lymphoblastic leukemia), CEM-SS (human T-lymphoblastic leukemia), and Kasumi-1 (human acute myeloid leukemia). The HEK293 and SC-1 cell lines were cultured in DMEM containing 10% fetal calf serum (FCS), 4 mM of *L* -glutamine, 100 U/ml of penicillin, and 100 µg/ml of streptomycin. The Jurkat, CEM-SS, and Kasumi-1 cell lines were cultured in RPMI-1640 containing 20% FCS, 4 mM of *L* -glutamine, 100 U/ml of penicillin, and 100 µg/ml of streptomycin. The cells were grown at 37°С in humid air containing 5% of СО _2_ . 

**Obtainment of pseudo-HIV-1 particles**

 HEK293 cells seeded in Petri dishes with a diameter of 100 mm in the amount of 3.0–3.5 × 10 ^6^  cells per dish 12–14 h prior to the transfection onset were used as packaging cells, in which the assembly of recombinant lentiviral (pseudo-HIV-1) particles occurs. 

DNA of the lentiviral vector containing the marker gene of green fluorescent protein (GFP) and the plasmids directing the synthesis of the proteins that are required for the formation of pseudo-HIV-1 particles were introduced into HEK293 cells via calcium phosphate transfection. The infectious pseudo-HIV-1 particles were collected 24 h following transfection with a 12 h interval [13].

The virus was titrated on HEK293 cells seeded to 24-well plates 24 h prior to infection. The level of cell fluorescence was measured on an Epics 4XL Beckman Coulter flow cytofluorimeter (USA) 48 h following the infection. The virus titer was calculated using the formula *T* = *NP* / *V* , where *N* is the amount of seeded cells, *P * is the share of the infected cells in the population, *V * is the amount of the added supernatant containing pseudo-HIV-1 particles, and *T*  is virus titer. The samples with virus titer of 5 × 0 ^5^ –5 × 10 ^6^ were used in this study. 

**Investigation of the viral activity of compounds**

 In order to assess the anti-HIV-1 activity, a solution of the analyzed substances in water or dimethylsulfoxide (DMSO, the final concentration in the medium was no higher than 0.1%), was added to the cells; after 2–8 h (depending on the inhibitor), the cells were infected with pseudo-HIV-1 particles. The relative level of infection was determined by flow cytofluorimetry on an Epics 4XL Beckman Coulter instrument (USA) 48 h following the infection. 

## RESULTS AND DISCUSSION

**Construction of pseudo-HIV-1 particles and using them to infect different eukaryotic cell lines**

**Table 1:** Antiviral activity of the investigated agents with respect to pseudo-HIV-1 particles pseudotyped with vesicular stomatitis virus G protein

Agent	Cell line	ID_50_, µm	
		Experimental data	Published data [20–29]
AZT	Jurkat	0.1 ± 0.01	0.004–0.1
	SC-1	0.08 ± 0.005	
	Kasumi-1	0.3 ± 0.02	
	CEM-SS	0.46 ± 0.05	
3TC	Jurkat	0.7 ± 0.05	0.02–0.35
	CEM-SS	0.85 ± 0.05	
d4T	Jurkat	7 ± 0.5	0.43–1.67
	SC-1	10 ± 0.5	
ddC	Jurkat	7 ± 0.5	0.067–0.316
	SC-1	5 ± 0.5	
ddI	Jurkat	> 20	1.79–12
	SC-1	> 20	
Nevirapine	Jurkat	0.1 ± 0.005	0.0072–0.22
	SC-1	0.15 ± 0.005	
	Kasumi-1	0.08 ± 0.005	
	CEM-SS	0.2 ± 0.01	
Non-nucleoside inhibitor of RT 1	Jurkat	0.95 ± 0.005	0.13
Non-nucleoside inhibitor of RT 2	Jurkat	0.08 ± 0.001	0.016
Non-nucleoside inhibitor of RT 3	Jurkat	0.085 ± 0.001	0.018
Raltegravir	Jurkat	0.009 ± 0.0005	0.0022–0.0037
	SC-1	0.006 ± 0.0005	
	CEM-SS	0.009 ± 0.0005	
L-731988	Jurkat	12 ± 0.1	1
	SC-1	8 ± 0.1	

 Efficiency of transduction of target cells with pseudo-HIV-1 particles, and thus the fluorescence level of the resulting transgenic cells, is the most significant parameter of a lentiviral system. This parameter depends on the structure of pseudoviral particles (the type of coat proteins) and the particular line of infected target cells. The transplantable human lymphoblastic cells Jurkat and CEM-SS (T-lymphoblastic leukemia contain specific HIV-1 receptors), Kasumi-1 (acute myeloid leukemia), and mouse embryonic fibroblasts SC-1 were used as target cells. 

Two types of pseudo-HIV-1 particles differing in coat proteins were obtained and subjected to study. Particles of the first type contain the HIV-1 coat protein gp160 (SUgp120 + TMgp41); particles of the second type contain the vesicular stomatitis virus (VSV) G protein. The use of particles of the first type resulted in a comparatively low transduction efficiency and a weaker fluorescence signal (the data are not presented) from the infected cells ( *Fig. 2* ). In the case of pseudo-HIV-1 particles carrying the VSV G protein, the share of infected cells and the level of expression of the green fluorescent marker protein (eGFP) were considerably higher ( *Fig. 2* ). Moreover, the particles pseudo-typed with the VSV G protein can be used to transfer marker genes to the cells with wide type and tissue specificity. This procedure enables one to perform the search for retroviruses affecting tissues other than blood. Therefore, pseudo-HIV-1 particles with the VSV G protein were the ones used in most experiments devoted to the study of the properties of inhibitors of HIV-1 reverse transcriptase and integrase. 

**Nucleoside inhibitors of HIV-1 reverse transcriptase**

**Fig. 4:**
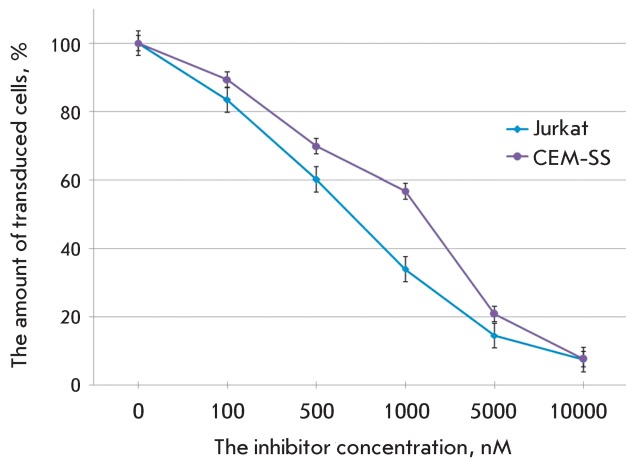
The action of 3TC on the transduction efficiency of the cell lines Jurkat and CEM-SS with pseudo-HIV-1 particles containing the envelope protein VSV-G. The level of transduction is shown with respect to the positive control.

**Fig. 5:**
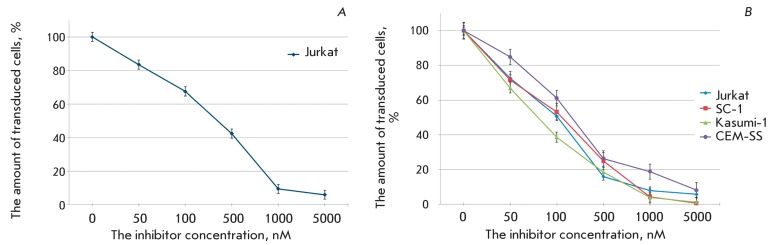
The action of nevirapine on the transduction efficiency of different cell lines with pseudo-HIV-1 particles containing the envelope protein gp160 (A) or envelope protein VSV-G (B). The level of transduction is shown with respect to the positive control.

 Modified nucleosides and nucleotides have found broad application in the therapy of various viral diseases, including the HIV-1 infection [3]. Their mechanism of action includes conversion of these compounds, in a cell, into the corresponding nucleoside triphosphates, which act as terminating substrates for viral DNA and RNA polymerases. The integration of nucleotides into the growing chain of viral DNA/RNA blocks viral replication and slows the spread of the infection. The first and most well-known anti-HIV-1 agent of this class is 3’-azido-3’-deoxythymidine (AZT), which can inhibit viral replication even at a nano-molar concentration. The antiviral activity of AZT was studied with respect to pseudo-HIV-1 particles carrying the HIV-1 coat protein gp160 or the VSV protein G on their surface. *Fig. 3* shows the effect of AZT on the efficiency of cell transduction with HIV-1-like particles containing reverse transcriptase, wild-type integrase, and HIV-1 coat protein gp160 ( *A* ) or the vesicular stomatitis virus G protein ( *B* ). It is clear that AZT suppresses the infection of eukaryotic cells with both types of pseudoviral particles, although the concentration of the particles is higher than that of infectious HIV-1 (Table) [ *20–22* ]. In the Jurkat cell culture, the activity of the agent was higher with respect to the particles pseudotyped with the VSV G protein. The antiviral activity of the nucleoside depended not only on the particle type, but also on the line of target cells. Thus, the maximum effect was observed on mouse SC-1 fibroblasts, whereas the minimum effect was observed when using CEM-SS cells. The reasons for these differences may be due to the different intracellular contents of nucleoside and nucleotide kinases [30], i.e., the enzymes required for the conversion of a nucleoside into the corresponding triphosphate, and the differences in the levels of expression of the specific transporters that are responsible for the transport of an agent into the cell, or its elimination [31]. 

Other well-known and commonly used antiretroviral agents are 2’,3’-dideoxy-3’-thiocytidine (3TC) and 2’,3’-2’,3’-didehydrothymidine (d4T); similar to AZT, they are nucleoside inhibitors of HIV-1 reverse transcriptase [3]. 3TC was synthesized in 1989 and certified for clinical use in 1995. It is currently being used in combination with other drugs. The efficiency of joint use of 3TC and AZT has been demonstrated. We assessed the antiviral activity of 3TC on Jurkat and CEM-SS cell lines ( *Fig. 4* ). Drug activity in our system was somewhat lower than recorded in published data [20, 24]. The activity of other nucleoside analogues, including d4T, was also lower for our system, in comparison with that shown for infectious HIV-1 ( *Table* ) [20, 21, 24]. 

**Non-nucleoside inhibitors  of HIV-1 reverse transcriptase**

**Fig. 6:**
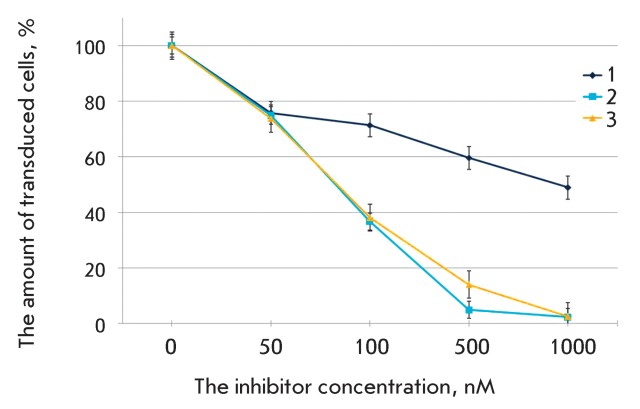
The action of non-nucleoside reverse transcriptase inhibitors of HIV-1 1, 2 and 3 on the transduction efficiency of the Jurkat cell line with pseudo-HIV-1 particles containing the envelope protein VSV-G. The level of transduction is shown with respect to the positive control.

**Fig. 7:**
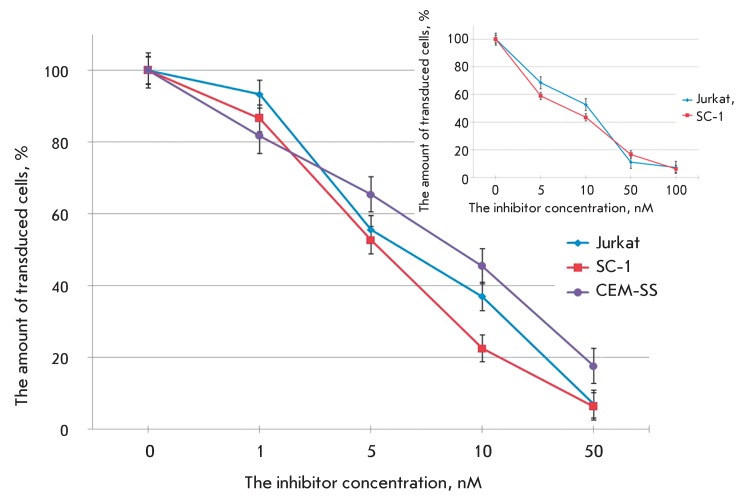
The action of HIV-1 integrase inhibitors raltegravir (main figure) and L-731988 (inset) on the transduction efficiency of different cell lines with pseudo-HIV-1 particles containing the envelope protein VSV-G. The level of transduction is shown with respect to the positive control.

 Nevirapine is the most commonly used non-nucleoside blocker of HIV-1 replication and reverse transcriptase inhibitor [3]. This compound was certified as a drug in 1996; at a concentration of 10 ^-8^ –10 ^-7^  М, it can slow the development of the HIV-1 infection in cells infected with the natural virus. We studied the ability of nevirapine to prevent the transduction of target cells using the above-described pseudo-HIV-1 particles. In similar fashion to AZT, nevirapine exhibited a higher antiviral activity towards pseudoviral particles carrying the VSV G protein on their surface ( *Fig. 5* ). Again, similarly to AZT, nevirapine was most efficient in the SC-1 fibroblast culture, and less efficient in the CEM-SS cell line. It should be emphasized that nevirapine activity in our system was comparable to its activity towards infectious HIV-1 [21, 25]. 

In addition to the commercially available drug nevirapine, we tested three non-nucleoside inhibitors (denoted with the numbers 1, 2, and 3) which were synthesized according to the procedure described in [27]. These compounds are N ^1^ -substituted uracils carrying benzophenone-oxyethyl (2 and 3) or benzyl-phenoxyethyl fragments (1). These compounds have been shown to possess high levels of anti-HIV-1 activity in a cell culture infected with the wild-type virus [27]. It was demonstrated that all three compounds can prevent the transduction of SC-1 cells with pseudo-HIV-1 particles with the VSV G protein; the activity of benzophenone-containing compounds (2 and 3) was considerably higher than that of the benzyl-phenoxyethyl-uracil derivative (1) ( *Fig. 6* ) and was comparable to that of nevirapine. The data obtained are in good correlation with the results of the study of these compounds in the infectious cell system ( *Table* ). 

**HIV-1 integrase inhibitors**

**Fig. 8:**
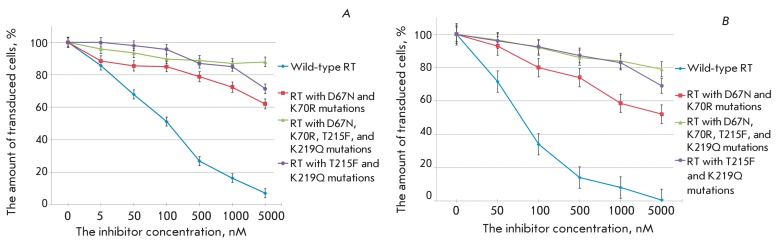
The action of AZT on the transduction efficiency of cells with pseudo-HIV-1 particles containing envelope protein VSV-G and reverse transcriptase of wild-type or mutant form, shown for the Jurkat (A) and SC-1 (B) cell lines. The level of transduction is shown with respect to the positive control.

**Fig. 9:**
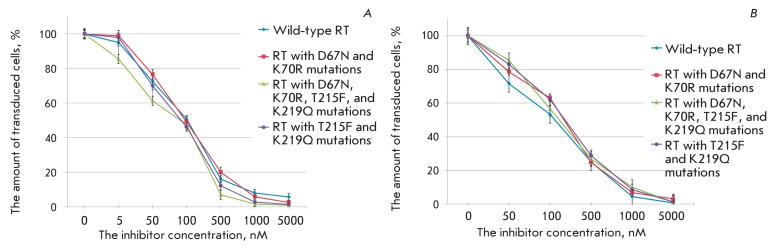
The action of nevirapine on the transduction efficiency of cells with pseudo-HIV-1 particles containing envelope protein VSV-G and reverse transcriptase of wild-type or mutant form, shown on the Jurkat (A) and SC-1 (B) cell lines. The level of transduction is shown with respect to the positive control.

 The commercially available drug raltegravir (certified for use in clinical practice in October 2007) and the well-known integrase inhibitor L-731988 were used to assess the potential of the designed system for screening integrase inhibitors [28]. Raltegravir and L-731988 block the second integration stage, the chain transfer, thus impeding integrase binding to cell DNA. The efficiency of cell transduction with pseudo-HIV-1 particles with wild-type integrase as a function of inhibitor concentration is shown in *Fig. 7* . It is clear that raltegravir activity is higher than that of L-731988 by approximately three orders of magnitude, a fact that correlates with the data obtained for the infectious system [28, 32]. A decrease in the amount of fluorescing cells in the presence of integrase inhibitors attests to the fact that an adequate integration of the synthesized DNA into the target cell genome takes place in the proposed pseudoviral system, and that pseudo-HIV-1 particles can indeed be used as a convenient tool for studying the antiviral activity of inhibitors of virus protease. 

**AZT-resistant pseudo-HIV-1 particles**

**Fig. 10:**
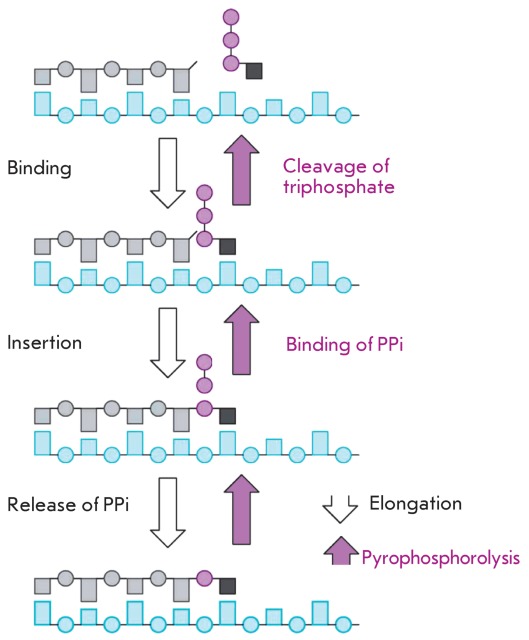
Mechanism of the pyrophosphorolysis reaction catalyzed by reverse transcriptase.

**Fig. 11:**
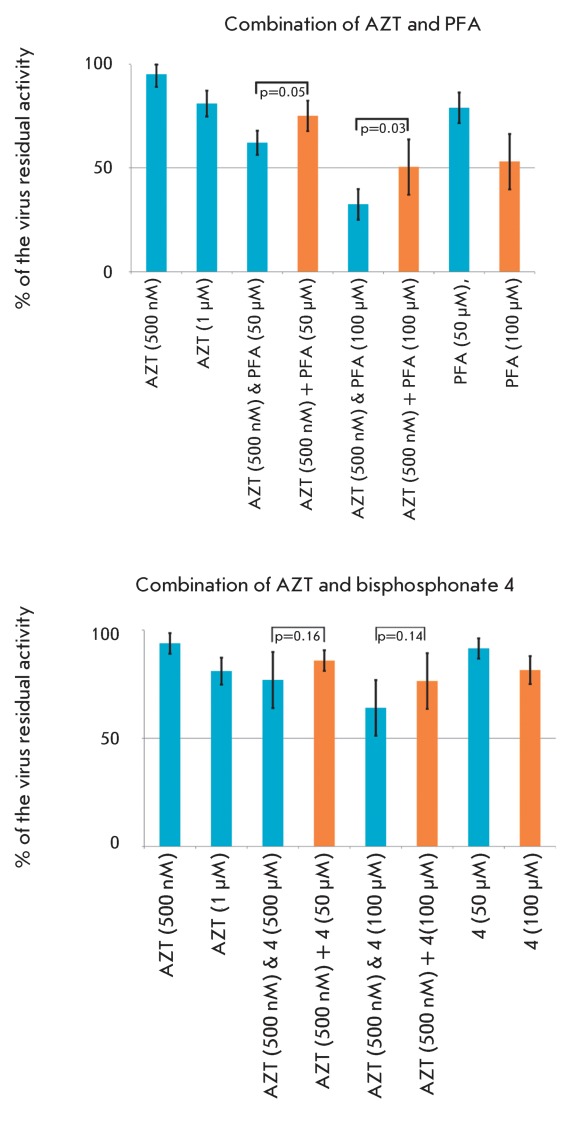
The suppression of viral transduction via the combination of pyrophosphorolysis inhibitors and AZT. The data are represented as the average value of a series of five experiments ± confidence interval (P ≤ 0.1). The values of the series “AZT &” were obtained by simultaneous application of inhibitors at the concentrations indicated in brackets. The values of the series “AZT +” were calculated by multiplication of the residual activity of individual inhibitors. Concentrations are indicated in brackets. p – value of Paired Two Sample T-test of Student for indicated values.

 The search for potential inhibitors of the replication of drug-resistant HIV-1 strains is a very important task. However, such studies are often hampered not only by the need to use an infectious virus that is dangerous for both personnel and the laboratory, but also by the complexity of obtaining strains that would be insensitive to this group of preparations. The proposed system allows one to easily construct variants of pseudo-HIV-1 particles that carry replication enzymes with the mutations determining their resistance to drugs. This fact was verified by constructing three types of pseudo-HIV-1 particles with the point substitutions D67N, K70R, T215F, and K219Q in reverse transcriptase, which are most typical of AZT-resistant HIV-1 strains [33, 34]. The antiviral activity of AZT was compared with that of these variants of pseudo-viral particles, demonstrating that AZT had a much weaker effect on the efficiency of transduction with mutant particles ( *Fig. 8* ). The decrease in the inhibiting effect correlated with an increase in the number of mutations (this effect was most clearly pronounced in SC-1 cells). Meanwhile, nevirapine, the non-nucleoside inhibitor of HIV-1 reverse transcriptase, retained its degree of activity towards all AZT-resistant types of pseudoviral particles ( *Fig. 9* ). This can be explained by the fact that the site of the binding to AZT is distant from the active site of the enzyme, which interacts with AZT triphosphate and contains all the aforementioned mutations. Thus, it is indeed pseudo-HIV-1 particles that allow one to study the ability of a substance to inhibit the drug-resistant forms of the virus. 

**Analogues of inorganic pyrophosphate**

 Another direction in approaches to the therapy of drug-resistant forms of HIV-1 consists in searching for compounds that would result in the recovery of virus sensitivity to the earlier used antiretroviral agents, when used in conjunction with such agents. The modern concept holds that HIV-1 resistance to nucleoside reverse-transcriptase inhibitors can be achieved using two alternative mechanisms, which include the emergence of the following mutations in reverse transcriptase: 

a) Mutations impeding the interaction between the enzyme and the corresponding nucleoside triphosphates (was described for 3TC) or:

b) Mutations facilitating the cleavage of the already-integrated terminating nucleotide from DNA during the pyrophosphorolysis reaction; after which synthesis of the growing DNA strand can continue (this mechanism is considered to be the major one for AZT) ( *Fig. 10* ). 

Thus far, a number of mimetic compounds of inorganic pyrophosphate capable of suppressing nucleotide cleavage upon pyrophosphorolysis have been described [35, 36]. One of these, foscarnet (PFA), has been successfully used in combination with AZT [37], a fact that supports the potential use of non-hydrolysable analogues of inorganic pyrophosphate in combination with nucleoside inhibitors in anti-AIDS therapy [37].
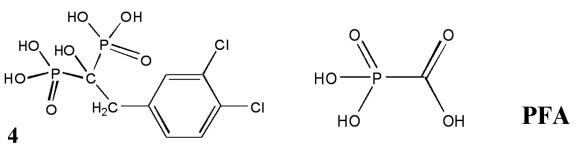

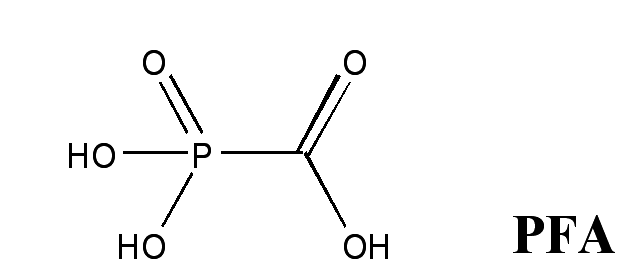


Derivatives of the hydroxymethylene diphosphonic acid, which are used in the therapy of bone-related diseases, are considered to be the most promising types of analogues of inorganic pyrophosphate. Contrary to foscarnet, this class of compounds do not behave as substrates in the pyrophosphorolysis reaction. Nevertheless, they efficiently inhibit the pyrophosphorolytic cleavage of AZT from DNA, which is catalyzed by HIV-1 reverse transcriptase [36]. It should also be noted that no data on their activity in cell systems have been published. In this study, foscarnet (PFA) and the analogue of inorganic pyrophosphate, biphosphonate  **4,** were selected to assess the adequacy of the proposed cell system and study this type of compounds. Among its analogues, the dichlorobenzyl derivative of methylene diphosphonic acid  **4** is the most active; it can suppress the cleavage of AZT monophosphate catalyzed by reverse transcriptase in the submicromolar concentration range [35]. The data on the joint action of azidothymine and the specified pyrophosphorolysis inhibitors are shown in *Fig. 11* . The degree of cell transduction inhibition with AZT-resistant pseudo-HIV-1 particles (carrying the point substitutions D67N, K70R, T215F, and K219Q in the reverse transcriptase) after the introduction of AZT combined with the selected pyrophosphorolysis inhibitor was determined in this experiment. The quantity of fluorescing cells in the individual presence of each of these substances was determined in the control experiment. 

A conclusion concerning the additivity of the action of AZT and pyrophosphate analogues was made by comparing the degree of inhibition in the presence of two substances and the product of the degrees of inhibition by each substance (which attests to the independence of their action). As can be seen in *Fig. 11* , foscarnet and biphosphonate  **4** suppressed cell infection with pseudoviral particles and provided a considerable and statistically significant enhancement of the action of AZT. Thus, the data obtained demonstrate, for the first time, that it is possible to recover the sensitivity of resistant forms of HIV-1 to nucleoside reverse transcriptase inhibitors in a cell culture. The data is also testament to the fact that analogues of inorganic pyrophosphate are promising agents for antiretroviral therapy. 

## CONCLUSIONS

A number of human and mouse cell lines were used to demonstrate that the described system for safe screening of potential HIV-1 replication inhibitors allows one to test the inhibitory activity of the compounds, whose action is directed both towards the reverse transcriptase and integrase of wild-type HIV-1 and towards their mutant forms corresponding to drug-resistant forms of the virus. It is important that the pseudo-HIV-1 particles used in this system are noninfectious. They are actually single-acting viruses (recombinant lentiviral vectors) that contain a complete set of viral enzymes ensuring the synthesis of the recombinant two-stranded DNA provirus and its integration into the genome of target cells. Next, the cell systems allow the expression of marker genes, which were integrated into the cell genome, within the recombinant genome of pseudo-HIV-1 particles.

The absence of a complete set of HIV-1 in this recombinant genome on one hand ensures safety when testing the efficacy of new anti-HIV-1 compounds and, on the other hand, enables to adequately assess the action of these compounds on HIV-1 reverse transcriptase and integrase in the cells infected (transduced) with pseudo-HIV-1 particles. The possibility of forming pseudo-HIV-1 particles containing mutant drug-resistant reverse transcriptase and/or integrase allows one to perform the screening of potential inhibitors of drug-resistant forms of HIV-1.

Pseudotyping of a pseudo-HIV-1 particle with coat proteins of retroviruses of a different nature (including HIV-1 coat protein gp160) and those of other enveloped viruses considerably broadens the possibilities of the screening system by enabling the infection of cells of different types, and it also enables testing of the inhibitors of virus penetration into the cell. Finally, this system allows one to study the HIV-1 protease inhibitors, although this was beyond the scope of the present work. 

## Abbreviations

HIV: human immunodeficiency virus
RT: reverse transcriptase
VSV: vesicular stomatitis virus
eGFP: enhanced green fluorescent protein
AZT: 3-azido-3- deoxythymidine
IC_50_: half maximal (50%) inhibitory concentration (IC) of a substance
